# Retracted: Measures of Adiposity and Risk of Testing Positive for SARS-CoV-2 in the UK Biobank Study

**DOI:** 10.1155/2022/9848453

**Published:** 2022-03-18

**Authors:** Journal of Obesity

*Journal of Obesity* has retracted the article titled “Measures of Adiposity and Risk of Testing Positive for SARS-CoV-2 in the UK Biobank Study” [1]. Following the publication of the article, the authors identified that there was a mistake in the SAS code that resulted in the age of the majority of patients being misclassified as one year older in Table 3.

A revised Table 3 is provided by the author as follows.

With the revised data, the minimum age of participants is 1 year younger and the median also decreased (from 71 (62 to 77) to 70 (61 to 76)). This increased the sample size in individuals under 65 and decreased the sample size in those over 65. Overall, there were slight changes to the effect estimates and confidence intervals in Table 3. It should be noted that the significance has changed for high waist circumference (HWC) in those over 65 years of age, moving from a *p* value reported as less than 0.05 to 0.052 when body mass index is examined as a continuous variable and 0.055 when body mass index is treated as a nominal variable.

The conclusions in the article are therefore no longer supported by the data, and the article is being retracted with the agreement of the editorial board.

The authors agree with this retraction.

## Figures and Tables

**Table 3:** Association between measures of adiposity and testing positive for SARS-CoV-2 overall and stratified by older age.

Variable	Full population^†^	Under 65 years^†‡^ (range 49–64)	65 years of age or older^†^ (range 65–84)
Total number tested for SARS-CoV-2, *n* (%)	9,386	3,095 (33.0)	6,291 (67.0)
Tested positive for SARS-CoV-2, *n* (%)	1,577 (16.8)	646 (20.9)	931 (14.8)
*Demographics*			
Male	1.19 (1.08, 1.30)^*∗*^	1.15 (1.00, 1.32)	1.30 (1.15, 1.48) ^*∗*^
*Race*			
White	Ref	Ref	Ref
Asian	1.68 (1.38, 2.05) ^*∗*^	1.83 (1.40, 2.39) ^*∗*^	1.45 (1.03, 2.04) ^*∗*^
Black	1.79 (1.49, 2.15) ^*∗*^	1.59 (1.25, 2.02) ^*∗*^	1.95 (1.45, 2.61) ^*∗*^
Mixed	1.00 (0.59, 1.67)	0.62 (0.28, 1.37)	1.84 (0.98, 3.47)
Missing	1.29 (0.97, 1.73)	1.27 (0.88, 1.84)	1.33 (0.82, 2.15)
*Anthropometric measures*			
Body mass index categories (continuous)	1.07 (1.03, 1.12) ^*∗*^	1.10 (1.04 to 1.18) ^*∗*^	1.04 (0.98 to 1.10)
*Body mass index categories*			
Underweight/normal weight	Ref	Ref	Ref
Overweight	1.15 (1.03, 1.29) ^*∗*^	1.20 (1.01, 1.42) ^*∗*^	1.10 (0.94, 1.29)
Obesity class I	1.15 (1.00, 1.32) ^*∗*^	1.20 (0.97, 1.47)	1.07 (0.89, 1.28)
Obesity class II	1.40 (1.18, 1.67) ^*∗*^	1.44 (1.11, 1.87) ^*∗*^	1.32 (1.04, 1.67) ^*∗*^
Obesity class III	1.20 (0.94, 1.54)	1.45 (1.02, 2.10) ^*∗*^	1.02 (0.72, 1.44)
HWC	1.07 (0.96, 1.18)	0.95 (0.80, 1.14)	1.13 (1.00 to 1.28)
